# Histological complete response to a combined docetaxel/cisplatin/fluorouracil neoadjuvant chemotherapy for T4 stage gastric adenocarcinoma

**DOI:** 10.1186/1477-7819-12-150

**Published:** 2014-05-19

**Authors:** Ming gao Guo, Qi Zheng, Jian zhong Di, Zhe Yang

**Affiliations:** 1 Department of Surgery, the Six People’s Hospital of Shanghai, Shanghai Jiaotong University, Shanghai 200233, China

**Keywords:** Gastric cancer, neoadjuvant chemotherapy, docetaxel, cisplatin, fluorouracil

## Abstract

Local advanced gastric carcinoma has a very poor prognosis. When a T4 gastric carcinoma has invaded the surrounding tissues and organs, curative resection is unlikely. We present here a case of a 63-year-old woman with a T4 unresectable gastric adenocarcinoma. She underwent two 3-week cycles of docetaxel/cisplatin/fluorouracil chemotherapy, followed by radical gastric resection. Each cycle consisted of 75 mg/m^2^ docetaxel and 75 mg/m^2^ cisplatin on day 1, and 200 mg/m^2^ leucovorin and 500 mg/m^2^ fluorouracil on days 1 through 5. The patient exhibited a complete histologic response. Our results indicate that docetaxel/cisplatin/fluorouracil neoadjuvant chemotherapy is a promising method of treatment for advanced gastric cancer.

## Background

Gastric cancer is one of the most common malignancies and leading cause of cancer mortality worldwide, with an estimated 900,000 new cases and more than 700,000 deaths in 2006 alone [1,2]. Radical gastrectomy with extended lymphadenectomy is now recognized as the only potentially curative treatment. Patients who present with stage III or IV disease, however, are not eligible for curative resection and thus have an especially poor prognosis. Moreover, local recurrence or distant metastasis can develop in a short time even after curative resection [3]. Over the past decade, an increasing number of reports have shown that neoadjuvant chemotherapy may be useful for patients with advanced T- and N-category lesions, possibly resulting in downstaging of tumors, improved rate of primary tumor resection with negative surgical margins, and early treatment of micrometastatic disease. We report here a case of a patient with a T4 unresectable gastric adenocarcinoma who exhibited a complete histologic response after docetaxel-based neoadjuvant chemotherapy.

## Case presentation

**Figure 1:**
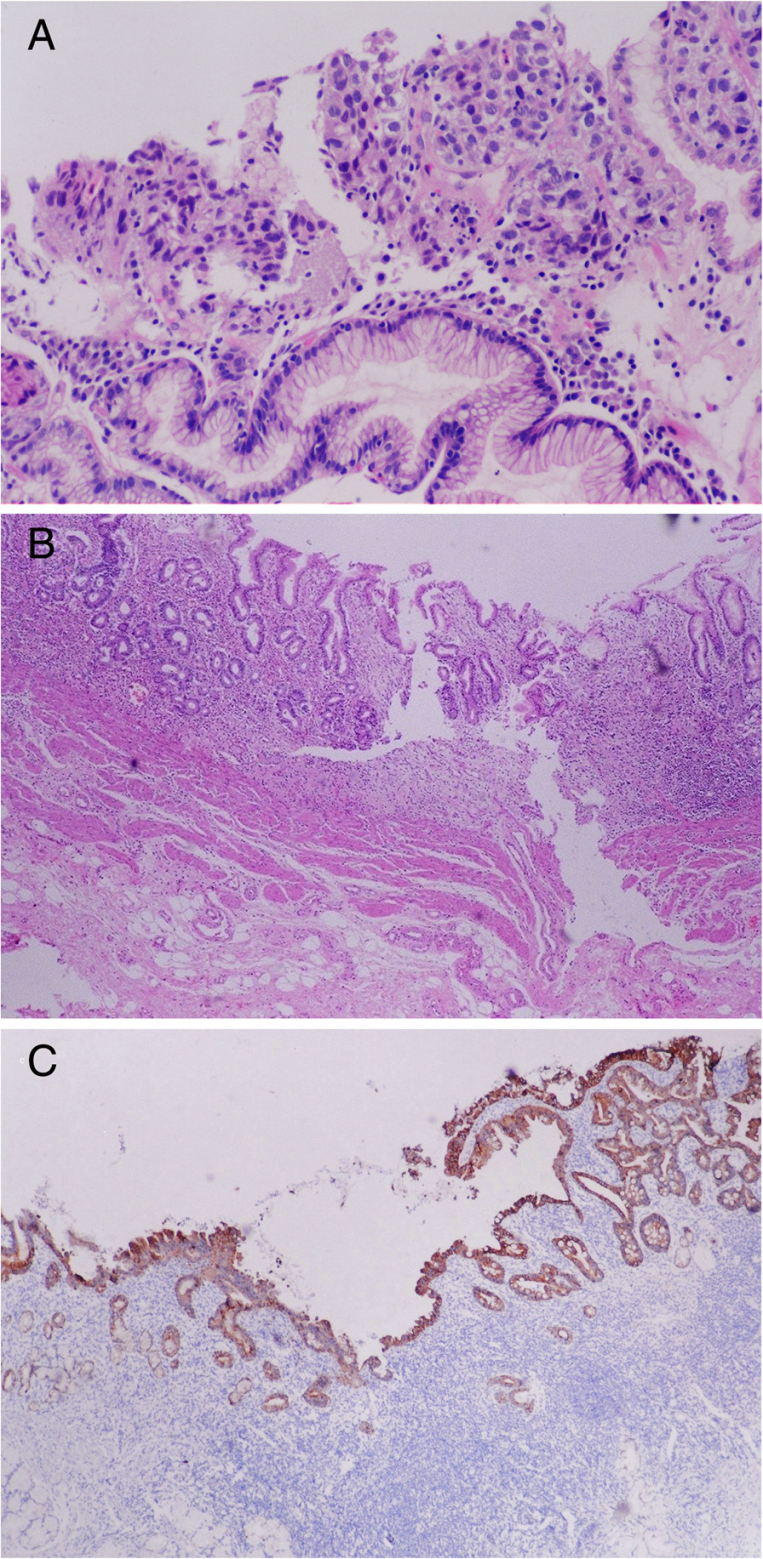
**Tumor and histopathological features. (A)** Biopsy indicated undifferentiated adenocarcinoma. **(B)** Microscopic examination of the excised specimen showed no remaining gastric adenocarcinoma cells and coverage of the lesion with regenerative mucosa. Gland degeneration was seen in the mucosa. Fibrosis and lymph follicle formation were observed in the gastric wall. **(C)** No gastric adenocarcinoma cell remnants were detected even with immunohistochemistry staining for CK20.

**Figure 2:**
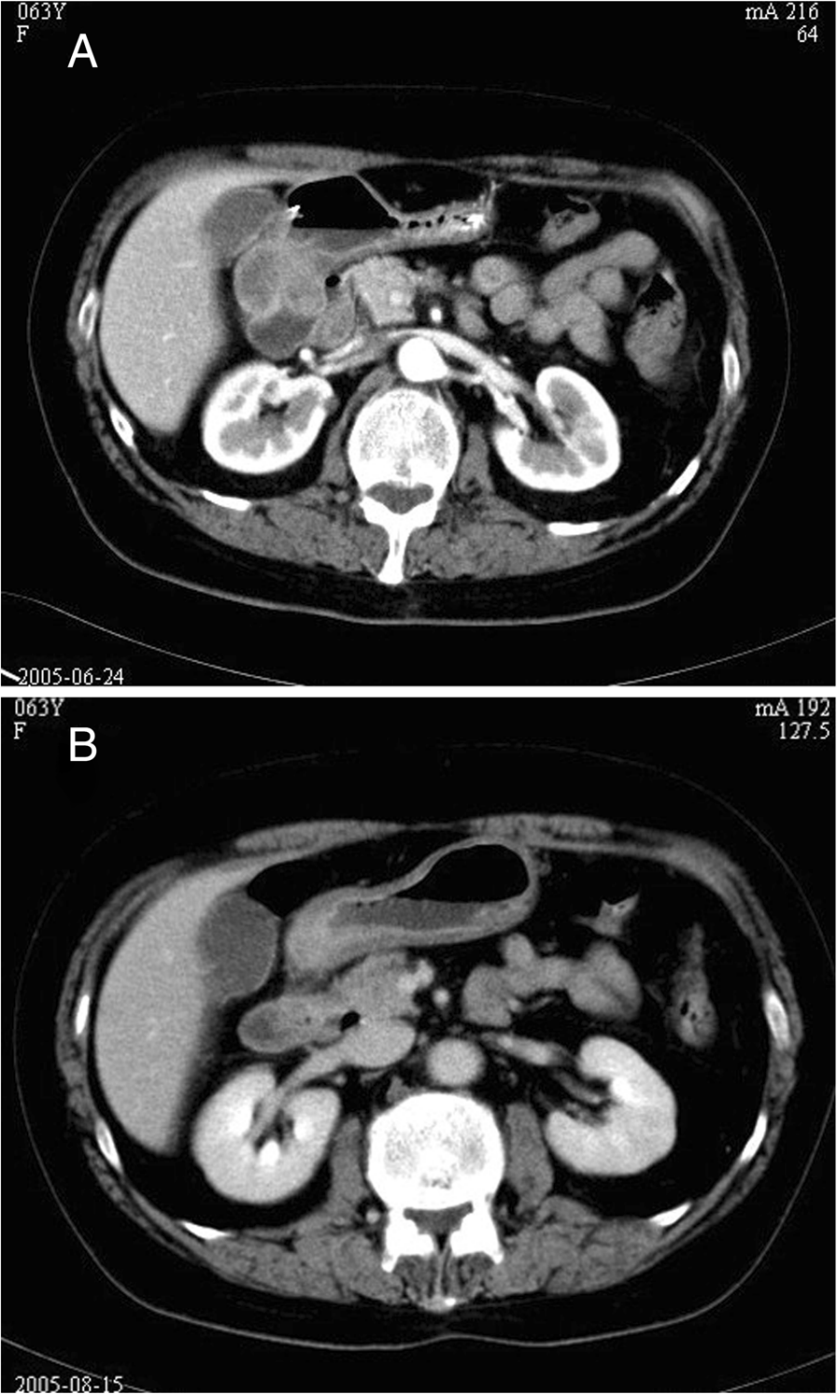
**Computed tomography of the gastric wall. (A)** Abdominal computed tomography (CT) scans revealed thickening of the gastric wall. Obliteration of the fat planes between the gastric tumor and adjacent organs indicated a T4 tumor. **(B)** CT scans after two courses of chemotherapy showed dramatic reduction of the primary lesion and clarification of the fat planes.

A 63-year-old woman presented to our local hospital because of epigastric pain. Endoscopic examination revealed a 45 × 52 mm irregular lesion in the lesser curvature of the antrum of the stomach. A biopsy indicated undifferentiated adenocarcinoma (Figure 1A). Abdominal computed tomography (CT) showed thickening of the gastric wall. Enlarged metastatic lymph nodes could not be found. Obliteration of the fat planes between the gastric tumor and adjacent organs indicated a T4 tumor [4,5] (Figure 2A). A diagnosis of advanced gastric cancer, T4N_X_M_0_, Stage IIIA or Iς, was made in accordance with the current International Union Against Cancer TNM staging system. Because of the difficulty of performing a radical operation, we recommended that the patient undergo chemotherapy.

Neoadjuvant chemotherapy was administered using docetaxel, cisplatin, fluorouracil, and leucovorin. On day 1, 75 mg/m^2^ docetaxel and 75 mg/m^2^ cisplatin were administered intravenously. On days 1 through final day of the cycle 500 mg/m^2^ fluorouracil and 200 mg/m^2^ leucovorin were administered. One cycle lasted 3 weeks, and two cycles were performed. Another CT scan was performed 2 weeks later. The results showed dramatic reduction of the primary lesion and clarification of the fat planes between the gastric tumor and adjacent organs (Figure 2B). Enlarged metastatic lymph nodes and ascites were not found.

Surgery was performed 4 weeks after neoadjuvant chemotherapy. No overt peritoneal metastases were found, and the gastric tumor was not obvious. The surgical procedure was distal partial gastrectomy with a 5-cm margin of safety. A D2 lymph node dissection according to the Japanese Research Society for Gastric Cancer (JRSGC) guidelines [6] was performed, and 21 lymph nodes were dissected. The Billroth II method was used for reconstruction of the alimentary tract.

Macroscopic examination revealed an irregular lesion extending to proprietary muscle, measuring 10 × 12 mm, in the lesser curvature of the excised gastric specimen. Microscopic examination indicated that no gastric adenocarcinoma cells remained and the lesion was covered with regenerative mucosa. Gland degeneration was seen in the mucosa. Fibrosis and lymph follicle formation were observed from the submucosa to the subserosa (Figure 1B). No gastric adenocarcinoma cell remnants were detected even with immunohistochemistry staining for CK20 (Figure 1C). No lymph node metastasis was found on pathologic examination. No additional chemotherapy was performed after surgery. Follow-up examination was conducted every 6 months for a total of 8 years. The patient remained healthy, with no tumor recurrence or metastasis. She was considered to have had a complete histologic response to treatment with neoadjuvant chemotherapy.

## Discussion

The 5-year survival rate for all gastric carcinoma patients undergoing surgery is only 20% to 30%. Local recurrence has been reported to be the main cause of treatment failure [7]. When T4 gastric carcinoma has invaded the surrounding tissues and organs, curative resection is achieved in only 33.3% of cases, although patients who undergo tumor resection (either curative or noncurative) have a significantly better survival rate than those who do not undergo resection [8].

During the last 20 years, preoperative chemotherapy has been used in an attempt to decrease the gastric tumor recurrence rate and increase the survival rate. The two main goals of neoadjuvant chemotherapy are downstaging the primary tumor to increase the chances of successful complete resection and destroying occult lymph nodes and distant metastases. Patients with clinical response to chemotherapy have significantly better prognoses than those without clinical response, particularly when complete (R0) resection is performed [9-11].

Several chemotherapy regimens have been developed for the treatment of advanced gastric cancer. Superior results have been achieved with epirubicin and cisplatin plus continuous-infusion fluorouracil (ECF). A phase III randomized trial showed that, compared with fluorouracil/doxorubicin/methotrexate, ECF showed a better overall response rate (ORR) (45% versus 21%), a longer median time to progression (TTP) (7.4 versus 3.4 months), and a longer overall survival (OS) (8.9 versus 5.7 months) [12]. Therefore, the investigators proposed ECF as a standard therapy [12,13]. More recently, several new agents have emerged, including taxanes, irinotecan, and oxaliplatin. Docetaxel was the first agent tried in advanced gastric cancer and showed better activity than the alternatives. Used as a single agent, docetaxel has been reported to have response rates between 17% and 29% [14-16]. Combined with other agents, paclitaxel has also shown promise [17,18]. The combination regimens, including paclitaxel/5-fluorouracil (5-FU)/cisplatin, resulted in response rates as high as 53%, with a median survival of 7 to 14 months [19-23]. The docetaxel/cisplatin/fluorouracil (DCF) regimen showed efficacy comparable to that of other regimens of cisplatin or anthracycline combinations. In the V325 Study Group, Van Cutsem *et al*. reported that adding docetaxel to cisplatin and fluorouracil (CF) significantly improved TTP, survival, and response rate in gastric cancer patients [22]. In a randomized phase II trial of the Swiss Group for Clinical Cancer Research, Roth *et al*. compared the therapy result of DCF with those of docetaxel and cisplatin (DC) and ECF. They reported an ORR of 25.0% (13 to 41%) for ECF, 18.5% (9 to 34%) for DC, and 36.6% (23 to 53%) for DCF (n = 119). Median overall survival times were 8.3, 11.0, and 10.4 months for ECF, DC, and DCF, respectively [23].

Hematotoxicity was the main adverse effect in docetaxel trials for advanced gastric cancer. Roth *et al*. reported a high incidence of febrile neutropenia associated with the DCF regimen, which occurred in 41% of patients, compared with only 18% and 21% of patients in the ECF and DC arms, respectively [23]. After the dose of docetaxel was reduced, the incidence of febrile neutropenia declined. According to the literature, 75 mg/m^2^ docetaxel combined with CF is well-tolerated, and this dose of docetaxel is most frequently proposed for treatment of gastric cancer [24-26]. Moreover, a higher incidence of febrile neutropenia did not translate to a higher mortality rate. This toxicity was acceptable, and the neutropenia was easily managed with granulocyte colony-stimulating factor (G-CSF). Interestingly, the higher incidence of toxicity did not appear to affect quality of life and clinical benefits, which were significantly greater with the DCF regimen, possibly because of the higher antitumor activity of DCF [22].

The histologic tumor regression grade is an objective measure of the effects of neoadjuvant chemotherapy. The overall rate of complete clinical response to DCF for advanced gastric tumor is 2 to 4.9% [22,23]. The rate of complete pathologic response, however, has been unknown.

Rapid tumor reduction before curative surgery is an important indicator of the success of neoadjuvant chemotherapy. Roth *et al*. reported that a DCF regimen resulted in a shorter median time to treatment response (TTR) than an ECF regimen (median TTR 1.6 versus 3.0 months) [23]. The patient with the T4 gastric tumor described here exhibited a complete pathologic response to DCF chemotherapy in 10 weeks. These results indicate that DCF neoadjuvant chemotherapy is a promising method of treating advanced gastric cancer.

## Conclusions

In conclusion, we observed a case of locally advanced gastric carcinoma, which exhibited a complete histologic response after neoadjuvant chemotherapy; the patient survived for more than 5 years. According to our experience, DCF neoadjuvant chemotherapy is a promising method of treatment for advanced gastric cancer.

## Consent

Written informed consent was obtained from the patient for the publication of this report and any accompanying images.

## Abbreviations

CF: cisplatin/fluorouracil; CT: computed tomography; DC: docetaxel/cisplatin; DCF: docetaxel/cisplatin/fluorouracil; ECF: epirubicin/cisplatin/fluorouracil; ORR: overall response rate; G-CSF: granulocyte colony-stimulating factor; TTP: time to progression; TTR: median time to treatment response.

## Competing interests

Ming gao Guo and the other co-authors have no competing interest.

## Authors’ contributions

MGG, QZ, JZD, ZY participated in the treatment of the patient. All authors read and approved the final manuscript.
